# Improvement of a delayed swallowing reflex following treatment for advanced head and neck cancer

**DOI:** 10.1186/s41199-020-00055-5

**Published:** 2020-06-02

**Authors:** Akira Ohkoshi, Kengo Kato, Takenori Ogawa, Ayako Nakanome, Ryo Ishii, Yukio Katori

**Affiliations:** Department of Otorhinolaryngology, Head and Neck Surgery, Tohoku University Graduate School of Medicine, 1-1 Seiryo-cho, Aoba-ku, Sendai, 980-8575 Japan

**Keywords:** Head and neck cancer, Swallowing disorder, Latency time of the swallowing reflex

## Abstract

**Background:**

The latency of the swallowing reflex is an important factor causing dysphagia in head and neck cancer patients. Although there are many reports comparing voluntary swallowing function before and after treatment, few studies have focused on the latency of the swallowing reflex, which is a risk factor for pneumonia due to silent aspiration. The aim of this retrospective study was to clarify the changes in the latency of the swallowing reflex before and after treatment.

**Methods:**

The latency of the swallowing reflex was quantified using the time from the injection of 1 ml of distilled water into the pharynx through a nasal catheter to the onset of swallowing.

**Results:**

The latency time of the swallowing reflex was significantly decreased 3 months after treatment compared to before treatment. A significant reduction was also observed in patients with pharyngeal cancer who underwent chemoradiation therapy.

**Conclusions:**

This retrospective study showed that a delayed swallowing reflex improved with treatment in advanced head and neck cancer patients.

**Trial registration:**

The Institutional Review Board of Tohoku University Hospital (Number 2014–1-274).

## Background

Advanced head and neck cancer and its treatment cause swallowing disorders that reduce patients’ quality of life and decrease survival [1, 2]. The mechanisms of dysphagia resulting from treatment of advanced head and neck cancer are various, such as reduced tongue base retraction, reduced laryngeal elevation, cricopharyngeal dysfunction, and a delayed swallowing reflex [3, 4]. Of the various mechanisms, both a delayed swallowing reflex and reduced elevation of the larynx are reported to be independent risk factors for aspiration pneumonia in head and neck cancer patients [5]. A delayed swallowing reflex may causes silent aspiration of oropharyngeal secretions, which is also known to be a risk factor for pneumonia in older people [6]. Although there are many reports of voluntary swallowing examined by videofluorography, the gold standard method for evaluation of dysphagia, or by flexible videoendoscopy, first reported in 1988 by Langmore and colleagues, few studies have examined silent aspiration due to attenuation of the swallowing reflex in head and neck cancer patients [7, 8]. The latency time of the swallowing reflex can be measured easily by bolus injection of a small amount of distilled water into the pharynx through a nasal catheter [9]. Head and neck cancer treatment, including both surgery and chemoradiation may result in a delayed swallowing reflex [10, 11]. Whereas direct invasion of tumor to the pharynx causes a delayed swallowing reflex, the disappearance of tumor with treatment may improve swallowing function [12, 13]. Although there are many reports comparing swallowing function before and after treatment, few studies have focused on the latency of the swallowing reflex [14, 15]. Thus, a retrospective study of patients with advanced head and neck cancer was conducted to clarify the changes of the latency of the swallowing reflex from before to after treatment.

## Methods

### Patient selection

This retrospective study was performed in accordance with the Helsinki Declaration and approved by The Institutional Review Board of Tohoku University Hospital (Number 2014–1-274). All patients who received treatment, including surgery and radiation therapy with or without chemotherapy, for advanced head and neck cancer and whose latency of the swallowing reflex was assessed both before and 3 months after treatment at the Department of Otolaryngology-Head and Neck Surgery of Tohoku University Hospital between April 2014 and March 2019 were included. Patients who underwent total laryngectomy at the same time or had recurrence within 3 month after treatment were excluded. The assessments were done at 3 month after completion of adjuvant treatment in patients who received post-operative therapy. In this study, 116 patients have received treatment for advanced head and neck cancer with laryngeal preservation, of which 73 patients have assessed the latency of the swallowing reflex before treatment. Finally, 53 patients have received the assessment both before and after treatment. Assessment of the latency of the swallowing reflex was performed before flexible endoscopic evaluation of swallowing, which was performed as standard practice for all patients with advanced head and neck cancer after they provided their written, informed consent.

### Assessment of the latency of the swallowing reflex

Individual latency time of the swallowing reflex was assessed before and 3 months after treatment. The swallowing reflex was induced by a bolus injection of 1 ml of distilled water into the pharynx through an 8-Fr nasal catheter. Before injection, the catheter was positioned below the soft palate. The subjects were unaware of the actual injection. Swallowing was identified by visual observation of characteristic laryngeal movement. The latency of the swallowing reflex was quantified using the time from the injection to the onset of swallowing, and the average of 3 measurements was used in the analysis [16]. According to previous research, the latency time of the swallowing reflex is 1.2 ± 0.1 s in healthy control subjects, 5.2 ± 0.6 s in older people with dementia, and 12.5 ± 3.0 s in older people with aspiration pneumonia [17]. Because a previous study treated patients with a latency time less than 3 s as a low-risk group for pneumonia, patients with a latency time greater than 3 s were defined as having a delayed swallowing reflex in this study [9].

### Statistical analysis

Differences were evaluated for significance using the paired *t*-test, Wilcoxon rank-sum test, or Fisher’s exact test. Values are presented as means ± SD.

## Results

A total of 53 patients (37 men and 16 women) were enrolled in this study, with an age range of 18–82 (median 65) years. The characteristics of the patients are shown in Table 1. There were 28 patients with oral cancers and 25 with pharyngeal cancers, including 17 oropharyngeal and 8 hypopharyngeal cancers. Forty-four patients were clinical stage IV, and 9 patients were stage III. Twenty-three patients underwent surgery, 22 patients received chemoradiation, 6 patients received both surgery and chemoradiation, and the other 2 patients received radiation without chemotherapy.

**Table 1:** Patients’ characteristics

Number of patients	53
Age (y) (median)	18–82 (65)
Sex (male/female)	37 / 16
Primary site	
Oral	28
Pharynx	25
Oropharynx	17
Hypopharynx	8
Clinical stage	
III	9
IV	44
Treatment	
Surgery	23
Chemoradiation	22
Surgery + Chemoradiation	6
Radiation	2

**Fig. 1:**
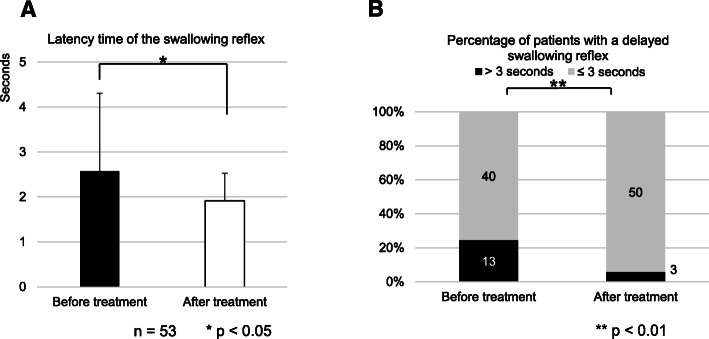
Latency of the swallowing reflex before and 3 months after treatment. Asterisks indicate significant differences between before and after treatment. The error bars indicate standard deviation. **a** Comparison of the latency time of the swallowing reflex before and 3 months after treatment (*n* = 53, *p* < 0.05, paired *t*-test). **b** Comparison of the percentage of patients with a delayed swallowing reflex before and 3 months after treatment (*p* < 0.01, Fisher’s exact test). Time over 3 s was defined as a delayed swallowing reflex

To evaluate whether the treatment of head and neck cancer changes the swallowing reflex, the latency time of the swallowing reflex was compared before and 3 months after treatment. The latency time of the swallowing reflex was significantly decreased at 3 months after treatment (1.91 ± 0.61 s) compared to before treatment (2.57 ± 1.73 s, *p* = 0.012, paired *t*-test, Fig. 1a). The percentage of patients with a delayed swallowing reflex was also significantly decreased at 3 months after treatment (3/53, 5.6%) compared to before treatment (13/53, 24.5%, *p* = 0.0073, Fisher’s exact test, Fig. 1b).

**Fig. 2:**
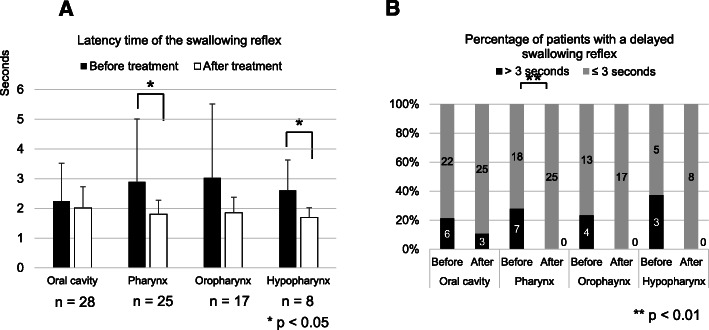
Latency of the swallowing reflex before and 3 months after treatment among the sites of head and neck cancer. Asterisks indicate significant differences between before and after treatment. The error bars indicate standard deviation. **a** Comparison of the latency time of the swallowing reflex between before and 3 months after treatment in cancers of the oral cavity (*n* = 28), pharynx (*n* = 25, *p* < 0.05, Wilcoxon rank-sum test), oropharynx (*n* = 17), and hypopharynx (*n* = 8, *p* < 0.05, Wilcoxon rank-sum test). **b** Comparison of the percentage of patients with a delayed swallowing reflex between before and 3 months after treatment in patients with cancers of the oral cavity, pharynx (*p* < 0.01, Fisher’s exact test), oropharynx, and hypopharynx. Time over 3 s was defined as a delayed swallowing reflex

Next, in order to examine the difference among the sites of head and neck cancer, the 53 patients were divided into those with cancers of the oral cavity (*n* = 28) and the pharynx (*n* = 25), which was further subdivided into oropharynx (*n* = 17) and hypopharynx (*n* = 8). Whereas there was no significant difference between before and 3 months after treatment in patients with the cancers of the oral cavity (2.24 ± 1.27 to 2.01 ± 0.71 s, *p* = 0.98, Wilcoxon rank-sum test), a significant reduction of the latency time of the swallowing reflex was observed in patients with cancers of the pharynx (2.89 ± 2.11 to 1.80 ± 0.46 s, *p* = 0.014, Wilcoxon rank-sum test, Fig. 2a). No significant reduction of the latency time of the swallowing reflex was observed in patients with cancers of the oropharynx (3.02 ± 2.48 to 1.85 ± 0.52 s), but it was observed in those with cancers of the hypopharynx (2.61 ± 1.01 to 1.88 ± 0.23 s, *p* < 0.05, Wilcoxon rank-sum test, Fig. 2a). On the other hand, the percentage of patients with a delayed swallowing reflex was not significantly changed in patients with cancers of the oral cavity (6/28, 21.4% to 3/28, 10.7%, *p* = 0.30, Fisher’s exact test), whereas a significant reduction was observed in patients with cancers of the pharynx (7/25, 28.0% to 0/28, 0%, *p* = 0.0096, Fisher’s exact test, Fig. 2b). However, no significant reduction of the percentage of patients with a delayed swallowing reflex was observed in patients with cancers of the oropharynx or hypopharynx (Fig. 2b).

**Fig. 3:**
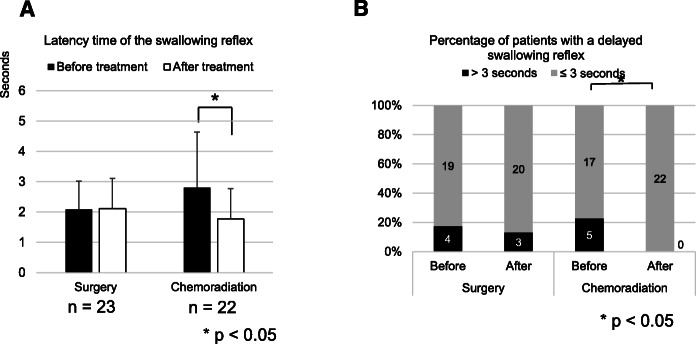
Latency of the swallowing reflex before and 3 months after surgery or chemoradiation. Asterisks indicate significant differences between before and after treatment. The error bars indicate standard deviation. **a** Comparison of the latency time of the swallowing reflex between before and 3 months after surgery (*n* = 23) or chemoradiation (*n* = 22, *p* < 0.05, Wilcoxon rank-sum test). **b** Comparison of the percentage of patients with a delayed swallowing reflex between before and 3 months after surgery or chemoradiation (*p* < 0.05, Fisher’s exact test). Time over 3 s was defined as a delayed swallowing reflex

Next, in order to examine the difference among treatments, the patients were divided into those treated with surgery (*n* = 23) and those treated with chemoradiation (*n* = 22). Patients who underwent both surgery and chemoradiation (*n* = 6) and those who underwent radiation without chemotherapy (n = 2) were excluded because of their small numbers. Whereas there was no significant difference between before and 3 months after treatment in the patients who underwent surgery (2.08 ± 0.93 to 2.10 ± 0.72 s, *p* = 0.68, Wilcoxon rank-sum test), significant reduction of the latency time of the swallowing reflex was observed in the patients who underwent chemoradiation (2.80 ± 1.83 to 1.76 ± 0.45 s, *p* = 0.025, Wilcoxon rank-sum test, Fig. 3a). On the other hand, the percentage of patients with a delayed swallowing reflex was not significantly changed in the patients who underwent surgery (4/23, 17.3% to 3/23, 13.0%,*p* = 1.00, Fisher’s exact test), whereas a significant reduction was observed in the patients who underwent chemoradiation (5/22, 22.7% to 0/22, 0%,*p* = 0.048, Fisher’s exact test, Fig. 3b).

## Discussion

This study demonstrated that a delayed swallowing reflex improved after treatment in advanced head and neck cancer patients. A delayed swallowing reflex is known to be an independent risk factor for aspiration pneumonia in head and neck cancer patients [5]. Both invasion of the tumor into the pharynx and cancer treatment including surgery and chemoradiation are known to cause a delayed swallowing reflex due to decreased pharyngeal sensation [10–12]. Although there are many reports comparing swallowing function, such as laryngeal elevation or penetration-aspiration, before and after treatment, few studies have focused on the latency of the swallowing reflex. To the best of our knowledge, this is the first report showing significant improvement in the delayed swallowing reflex after treatment in head and neck cancer patients. Despite an increased rate of dysphagia in patients after treatment due to reduced tongue base retraction, reduced laryngeal elevation, cricopharyngeal dysfunction, and so on, some patients reported improved swallowing function [18, 19]. In addition to disappearance of tumor, the improvement of a delayed swallowing reflex shown in the present study may contribute to recovery from dysphagia in such cases. In the present study, improvement of a delayed swallowing reflex was observed only in patients with pharyngeal cancer or who received chemoradiation. Because most patients (22 of 25 patients) with pharyngeal cancer received chemoradiation, the difference between the two groups was considered to be small in the present study. Since chemoradiation for pharyngeal cancer causes various sensory disorders with mucositis, such as pharyngeal pain, xerostomia, and dysgeusia, the improvement of a delayed swallowing reflex shown in the present study may have some relationship with pharyngeal hypersensitivity following radiation pharyngitis [20, 21]. On the other hand, no improvement of the swallowing reflex was observed in patients with oral cancer or those who underwent surgery in the present study. Because the swallowing reflex was induced by injection of water into the pharynx in the present study, oral cancer without direct invasion into the pharynx or surgery without manipulation of the pharynx was thought to have little effect on the swallowing reflex.

In contrast to the results of the present study, many previous reports have shown that chemoradiation causes a delay of the swallowing reflex [4, 5]. The contradiction can be explained by the fact that many previous reports examined voluntary swallowing, but the present study focused on spontaneous swallowing in which the subjects were unaware of the actual injection of distilled water into the pharynx. The spontaneous swallowing reflex is reported to mediated by substance P, which is also known to be upregulated by capsaicin [6, 9]. Interestingly, radiation therapy is reported to induce an early increase in substance P and produce an increased cough reflex [22]. However, substance P is also reported to decrease with long-term follow-up [23]. In the present study, the swallowing reflex was evaluated at only one point following treatment, 3 months after treatment; long-term follow-up might show the return of a delayed swallowing reflex.

The present study has several limitations. First, this retrospective study did not consider oral food intake or swallowing function other than the latency of the swallowing reflex. As in many other reports, despite the improvement of the delayed swallowing reflex, oral food intake of the patients in the present study did not improve after treatment (data not shown). This is thought to be because oral food intake is affected by many factors other than the latency of the swallowing reflex, such as prophylactic nutrition tube placement, xerostomia, and dysgeusia [24]. Second, the latency of the swallowing reflex was measured at only one point following treatment, 3 months after treatment, in the present study. Although pharyngeal mucositis and pain have ceased at this point, hypersensitivity of the pharynx can be present and affect the results. Third, this study had a small sample size, and only the data of patients who underwent testing both before and 3 months after treatment were included, so that there was some selection bias. Moreover, this study included small numbers of pharyngeal cancer patients who underwent surgery. Surgery of the tongue base, which is well known to cause dysphagia, could have affected the results of the present study [25]. Despite these limitations, this study provides valuable information about the latency of the swallowing reflex before and after treatment in head and neck cancer patients. The result will be helpful for better management of patients with swallowing disorders, and further prospective evaluation of large numbers of patients at later time points after treatment may be of greater benefit.

## Conclusion

This retrospective study showed that a delayed swallowing reflex improved after treatment in advanced head and neck cancer patients.

## Data Availability

The datasets analyzed during the current study are available from the corresponding author on reasonable request.
